# Intelligent identification of natural gas pipeline defects based on improved pollination algorithm

**DOI:** 10.1371/journal.pone.0288923

**Published:** 2023-07-27

**Authors:** Yiqiong Gao, Zhengshan Luo, Yuchen Wanng, Jihao Luo, Qingqing Wang, Xiaomin Wang, Aorui Bi

**Affiliations:** 1 School of Management, Xi’an University of Architecture and Technology, Xi’an, Shanxi, China; 2 Ruixin College, Beijing Institute of Technology, Beijing, China; 3 School of Management Engineering, Huaiyin Institute of Technology, Huaian, Jiangsu, China; University 20 Aout 1955 skikda, Algeria, ALGERIA

## Abstract

As a natural gas pipeline approaches the end of its service life, the integrity of the pipeline starts failing because of corrosion or cracks. These and other defects affect the normal production and operation of the pipeline. Therefore, the identification of pipeline defects is critical to ensure the normal, safe, and efficient operation of these pipelines. In this study, a combination of adaptive adjustment based on conversion probability and Gaussian mutation strategy was used to improve the flower pollination algorithm (FPA) and enhance the search ability of traditional flower pollination. The adaptive adjustment of the transition probability effectively balances the development and exploration abilities of the algorithm. The improved flower pollination algorithm (IFPA) outperformed six classical benchmark functions that were used to verify the superiority of the improved algorithm. A Gaussian mutation strategy was integrated with IFPA to optimise the initial input weights and thresholds of the extreme learning machine (ELM), improve the balance and exploration ability of the algorithm, and increase the efficiency and accuracy for identifying pipeline defects. The proposed IFPA-ELM model for pipeline defect identification effectively overcomes the tendency of FPA to converge to local optima and that of ELM to engage in overfitting, which cause poor recognition accuracy. The identification rates of various pipeline defects by the IFPA-ELM algorithm are 97% and 96%, which are 34% and 13% higher, respectively, than those of FPA and FPA-ELM. The IFPA-ELM model may be used in the intelligent diagnosis of pipeline defects to solve practical engineering problems. Additionally, IFPA could be further optimised with respect to the time dimension, parameter settings, and general adaptation for application to complex engineering optimisation problems in various fields.

## 1. Introduction

Natural gas provides high-quality clean energy, and abundant reserves thereof are available, which is of great significance to environmental protection and the sustainable economic development of countries worldwide. Natural gas is one of the main energy sources in the world [1]. According to the International Energy Agency, a quarter of the total global energy demand is met by natural gas [2]. Pipelines are considered the most preferred and safe means of transporting natural gas [3]. However, the pipe body of a pipeline may develop defects because of the damage inflicted by the pipeline service environment, geological conditions, stray currents, failure of anti-corrosion coatings, or third-party damage, among other reasons [4]. These defects may cause the pipeline to leak, leading to disasters [5]. Consequently, the safety of pipelines is becoming increasingly important, particularly because they cover long distances [6].

Traditional methods for the identification of pipeline defects, such as a BP neural network, cannot identify the defect categories of natural gas pipelines efficiently and accurately owing to the long training and learning times and the many parameters required to train the neural network. The extreme learning machine (ELM), a new type of learning algorithm based on a neural network, has been widely used for classification, regression, clustering, and feature learning [7]. The ELM is based on a model powered by an algorithm and is widely applied. The algorithm of the ELM model has been optimised for various fields and for solving a variety of problems. Although the ELM offers faster learning under the premise of ensuring learning accuracy, it has a superior pan-China effect [8]. However, ELMs also have shortcomings such as insufficient prediction accuracy and low convergence accuracy [9]. Although the ELM method is widely used in other defect identification fields, it has not found widespread use in the field of pipeline defect identification.

In essence, an ELM uses an algorithm based on a feedforward neural network [10]. However, it is prone to overfitting and has poor prediction accuracy. Therefore, introducing intelligent optimisation algorithms is necessary for improving the learning and prediction abilities of ELMs [11]. Existing intelligent optimisation algorithms are roughly divided into two categories: traditional and heuristic [12]. Traditional optimisation methods are designed to solve clearly structured optimisation problems. In comparison, heuristic optimisation methods can intelligently and efficiently search for the global optimum, and they can solve technical problems that cannot be solved using the traditional methods [13]. After more than two decades of development, heuristic algorithms have matured to become the mainstream algorithms for intelligent optimisation [14].

Despite their advantages, heuristic algorithms must be developed further such that they are efficient and robust, because traditional optimisation algorithms have limited ability to solve large-scale, highly nonlinear, and non-differentiable problems. The development of intelligent and efficient algorithms is required for real-world optimisation intelligence [15]. In this regard, meta-heuristic algorithms are intelligent and have many advantages, such as being powerful, efficient, flexible, and reliable [16]. Swarm intelligence and evolutionary algorithms are two very useful types of algorithms capable of intelligent optimisation [17]. The swarm intelligence algorithm develops intelligent search strategies by modelling and simulating the collective foraging behaviour of gregarious insects or animals using very few rules [18]. This method is characterised by its simplicity, efficient optimisation, and robustness. Various types of intelligent algorithms include differential evolution, whale optimisation, ant colony, particle swarm, colony foraging optimisation, frog leaping, artificial bee colony, flower pollination, firefly, cuckoo, bat, wolf swarm, fireworks, contract network protocol, and spider monkey optimisation algorithms. The evolutionary algorithm is an adaptive heuristic search algorithm based on the idea of natural selection and genetic evolution, which replaces an entire population by generating new offspring using natural operators such as crossover and mutation [19]. These algorithms include genetic, differential evolution, immune, genetic programming, evolutionary programming, evolutionary strategy, cultural gene, and multi-objective evolutionary algorithms. The advantages of the differential evolution algorithm, a global optimisation algorithm based on swarm intelligence, are its few control parameters, simplicity, and facile implementation. However, it readily converges to local optima, has slow convergence, and has numerous control parameters [20–22]. The whale optimisation algorithm has the advantages of simple operation, few control parameters, and a strong ability to escape from local optima, and it has attracted the attention of researchers in the field of swarm intelligence optimisation. However, this method is problematic because of its low convergence speed and accuracy [23].

The flower pollination algorithm (FPA) is a new meta-heuristic swarm intelligence optimisation algorithm proposed by Yang (2012) [24], a scholar at the University of Cambridge, UK in 2012. It simulates the processes of cross-pollination and self-pollination of flowering plants. Compared with other algorithms, the FPA has the advantages of fewer hyperparameters, a simple structure, and facile implementation. Therefore, the algorithm has been widely used for multi-objective and function optimisation as well as to solve prediction problems [25]. The FPA also has shortcomings, such as its tendency to converge to local optima, slow convergence during the late stage of iteration, and poor optimisation accuracy [26].

In this study, we propose a model framework for identifying defects in pipelines. We propose an improved flower pollination algorithm (IFPA), which combines an adaptive method based on transformation probability and Gaussian mutation strategies to improve the performance of FPA. Its optimisation performance is attributed to, amongst others, the FPA and particle swarm optimisation (PSO). Furthermore, the IFPA can be used to optimise the extreme learning machine (ELM). Compared with ELM and FPA-ELM, using the IFPA-ELM algorithm can more accurately identify the different states of the pipeline, such as the normal state of the pipeline, pit defects and crack defect signals and so on.

## 2. Basic theories

The existing quality problems of long-haul natural gas pipelines in service and the influence of external environment will produce various types of defects. However, the nondestructive testing technology of pipeline can only preliminarily judge whether the pipeline has defects according to the original signal itself, and cannot quantitatively identify the characteristics of defect type, defect degree, defect size and shape. Although various algorithms can provide new methods for pipeline defect detection, insufficient data and unbalanced samples are common problems that affect the generalization ability and robustness of pipeline defect identification models. In order to solve the above problems, the improved pollination algorithm combined with the extreme learning machine model was used in this study to improve the identification accuracy of pipeline defects.In this section, the FPA algorithm, adaptive adjustment method, Gaussian mutation strategy ,and IFPA algorithm are introduced.

### 2.1. FPA

The FPA was first proposed by Yang in 2012 as a new meta-heuristic swarm intelligence algorithm to simulate the flower pollination process of flowering plants in nature [24]. Flower pollination algorithms have been widely used for function optimisation, feature selection, model prediction, signal and graph processing, economic scheduling and control, and path planning [27]. The pollination of flowering plants occurs via cross-pollination and self-pollination; in the FPA, the simulated cross-pollination performs the function of global optimisation, and the simulated self-pollination that of local optimisation. Using the transition probability *P*, the global and local search can be converted into each other to solve the balance problem [28]. The specific rules are as follows:

1. Cross-pollination is a global pollination process in which pollen is spread by pollen dispersers over a long distance in Levy flight, ensuring the most suitable pollination and reproduction.
2. Self-pollination is a local pollination process.
3. The constancy of a flower is the reproductive probability, which is proportional to the similarity of the two flowers involved.
4. The global and local pollination are switched with the transition probability $P\in \left[0,1\right]$. The pollination process is more inclined to self-pollination during the process of flower pollination owing to the influence of factors such as location and wind. Therefore, the transition probability *P* has a larger value for local pollination.

**Fig 1:**
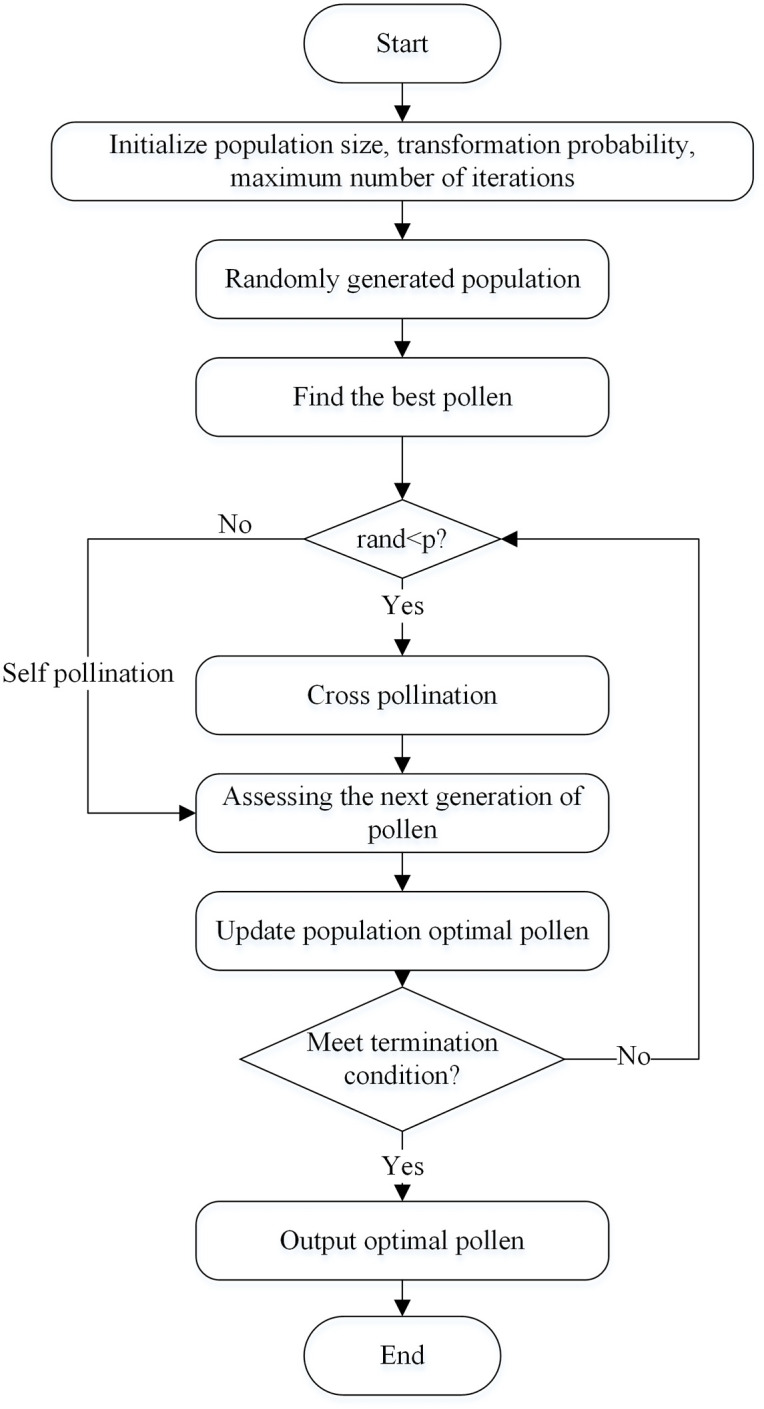
Flowchart of the FPA algorithm.

In the FPA, global and local pollination are the core of individual flower evolution. Establishing a mathematical model of its pollination process can lay a theoretical foundation for solving a series of complex optimisation problems. A flowchart of the algorithm is shown in Fig 1.

The global pollination process of the FPA is expressed as follows [29]:

$$x_{i}^{t+1}=x_{i}^{t}+\gamma L\left(x_{i}^{t}-g^{*}\right)$$ (1)

In Eq (1), $x_{i}^{t+1}$ and $x_{i}^{t}$ are the solutions of the pollen in the t+1^th^ and *t*^th^ generations, respectively; g^*^ is the global best position in the current population; γ is the scaling factor that controls the step size; L is the size of the random search step of the Levy flight [30]. and is calculated as follows:

$$L∼\frac{\lambda \Gamma \left(\lambda \right)\sin\left(\frac{\pi \lambda }{2}\right)}{\pi }\frac{1}{s^{1+\lambda }}\left(s\gg s_{0}≻0\right)$$ (2)

where Г(λ)is the standard gamma function, considering λ = 3/2, and s is the step size generated by the Mantegna algorithm.

In self-pollination, the equation for the updated position x^t^_i_ is as follows:

$$x_{i}^{t+1}=x_{i}^{t}+\varepsilon \left(x_{j}^{t}-x_{k}^{t}\right)$$ (3)

where x^t^_j_ and x^t^_k_ represent two random solutions of the population, and ε is a variable that obeys a uniform distribution in[0,1].

### 2.2. Adaptive adjustment based on transition probability

The transformation probability *P* is introduced into the FPA to prevent local optimisation resulting in premature convergence and to achieve a balance between the development and exploration abilities of the algorithm. *P* is the global pollination operation, and 1-*P* performs the local pollination operation, where *P* is a constant [31]. If the value of *P* is too large, the algorithm easily reaches the global optimum but does not easily converge; if the value of *P* is too small, it readily converges to a local optimum solution. Therefore, an adaptive adjustment method based on the transformation probability *P* is proposed:

$$P=0.8+0.2\times rand_{1}$$ (4)

Where rand_1_ is a random number between[0,1]. The initial conversion probability of cross-pollination and self-pollination was assumed *P* = 0.8.

In each iteration, the execution probability of the global and local search is updated adaptively, which effectively solves the balance problem between the development and exploration abilities of the algorithm, prevents the FPA from converging to local optima, and increases the convergence speed of the algorithm.

### 2.3. Adaptive adjustment based on gaussian mutation strategy

In FPA local pollination, the random number *ε* is in the range [0,1], and it controls the mutation probability of *i* pollen. If ε is too small, the position of the pollen before iteration is too close to that after iteration, resulting in slow convergence of the algorithm. If ε is too large, the position of the pollen is too poorly defined, and the jump step size is too large, which is not conducive for determining the optimal solution. Therefore, a Gaussian mutation strategy is employed because of the lack of a mutation mechanism in the local pollination process, making escape from a local optimum difficult [32]. Essentially, the Gaussian mutation strategy adds a random disturbance vector that obeys the Gaussian distribution to the original; thus, an individual to replace the original individual, in which case:

$$x_{i}=x_{i}+bN(0,1)x_{i}$$ (5)

In Eq 5, *b* = (*T* − *t*)/*T*, where *t* is the current number of iterations, and *T* is the maximum number of iterations. N(0,1) obeys the standard Gaussian distribution with u = 0 and σ^2^ = 1.

The convergence speed in the early stage of the computation by the algorithm can be improved by a larger Gaussian mutation vector, and in the later stage, a smaller Gaussian mutation vector may improve the convergence accuracy. In particular, when the algorithm converges to a local optimum in the later stage, a more appropriate global value exists near the current value. The Gaussian mutation vector can then be used to achieve the effect of escaping from the local optimum and detecting the fitness value of surrounding individuals. The local pollination process based on the Gaussian mutation strategy is identified as:

$$x_{i}^{t+1}=x_{i}^{t}+\varepsilon \left(x_{j}^{t}-x_{k}^{t}\right)+bN\left(0,1\right)x_{i}^{t\text{+}1}$$ (6)

### 2.4. Improved Flower Pollination Algorithm (IFPA)

The traditional FPA has the advantages of practicality, simplicity, and fewer parameters. Furthermore, gradient information is not necessary therein. However, while attempting to solve high-dimensional and multimodal optimisation problems, it cannot achieve the expected results. Therefore, an improved FPA is proposed, which, combined with the adaptive adjustment method of the transition probability and Gaussian mutation strategy, further improves the global and local search and optimisation capabilities of the FPA.

#### 2.4.1. Description of algorithm

**Fig 2:**
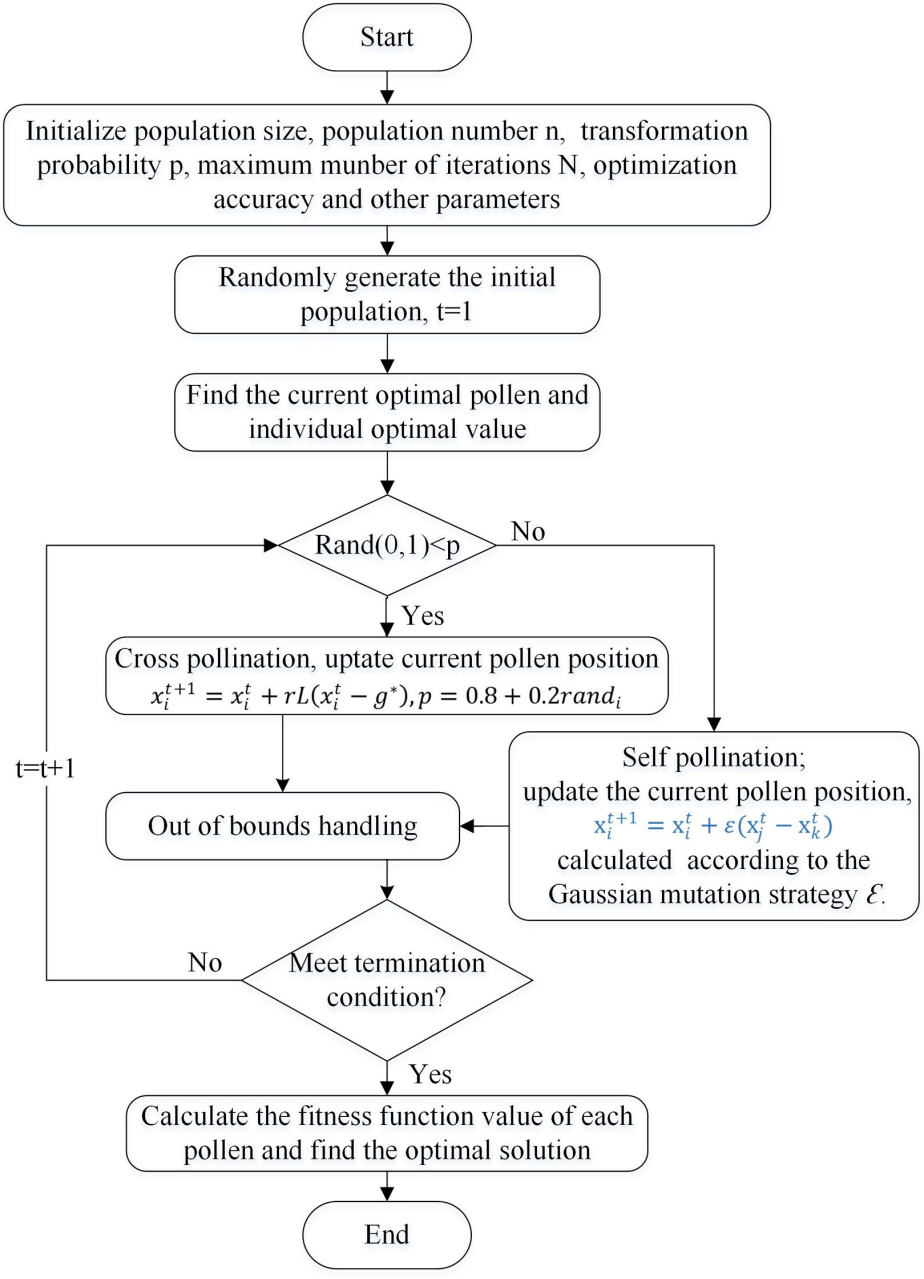
Flowchart of the IFPA algorithm.

This section mainly introduces the optimisation technology of the IFPA algorithm and proposes the IFPA algorithm, which uses the adaptive adjustment of the transition probability to enhance the global search and exploration abilities. The Gaussian mutation strategy is used to mutate the local solution, which effectively improves the convergence speed and accuracy of the algorithm. Thus, development and exploration are more optimally balanced by adaptively adjusting the transition probabilities and introducing Gaussian mutation strategies. Moreover, compared with the FPA, the IFPA also has the global pollination capability of Levy flight. A flowchart of the proposed IFPA is shown in Fig 2.

#### 2.4.2. Simulation test and analysis of result

The superiority of the IFPA is verified by comparing the classical optimisation algorithms, FPA, Spider monkey optimization (SMO) and African vulture optimization algorithm (AVOA). The IFPA algorithm is compared with three traditional algorithms. Six representative functions were selected from the literature [33] and the literature [34] as test functions to evaluate the performance of IFPA. Among them, f_1_–f_2_ are unimodal functions, and f_3_–f_10_ are multi-peak and multi-extremum functions. Using the same population size value of 20 for all algorithms, we ran 30 times independently, where the maximum number of iterations was 1000, and the search accuracy was 10^−5^. The optimal, worst, average value and standard deviation are used for evaluating the performance of the four algorithms. The parameters of the test function are shown in Table 1. All algorithms were run under Windows 7 environment, using Matlab 7.0 for programming.

**Table 1:** Parameters of the test function.

name	test function	Equation	minimum
Sphere	*f*_1_	$\sum_{i=1}^{n}x_{i}^{2}$	0
Rastrigrin	*f*_2_	$\sum_{i=1}^{n}\left[x_{i}^{2}-10\cos\left(2\pi x_{i}\right)+10\right]$	0
Ackley	*f*_3_	$-20\exp\left(-0.2\sqrt{\frac{1}{n}\sum_{i=1}^{n}x_{i}^{2}}\right)-\exp\left(\frac{1}{n}\sum_{i=1}^{n}\left(\cos2\pi x_{i}\right)\right)+20+e$	0
Griewank	*f*_4_	$1+\frac{1}{4000}\sum_{i=1}^{n}x_{i}^{2}-\overset{n}{\underset{i=1}{\Pi \cos\left(\frac{x_{i}}{\sqrt{i}}\right)}}$	0
Schaffer	*f*_5_	$\frac{\sin\sqrt[{2}]{x_{1}^{2}+x_{2}^{2}}-0.5}{{\left[1+0.001\left(x_{1}^{2}+x_{2}^{2}\right)\right]}^{2}}-0.5$	-1
Zakharov	*f*_6_	$\sum_{i=1}^{n}x_{i}^{2}+{\left(\frac{1}{2}\sum_{i=1}^{n}ix_{i}\right)}^{2}+{\left(\frac{1}{2}\sum_{i=1}^{n}ix_{i}\right)}^{4}$	0

The test values of the four optimisation algorithms in the six test functions and four test functions are compared, and the results are shown in Tables 2 and 3. The results show that the scores of the IFPA are higher than those of the FPA and traditional AVOA and SMO algorithms. The results therefore show that the proposed IFPA can more accurately optimise the test function to obtain the final optimisation result.

**Table 2:** Performance comparison of four optimization algorithms of reference function *f*_1_–*f*_6_.

Function	Dimension	Algorithm	Best value	Worst value	Average value	Standard deviation
*f*_1_	30	FPA	4.34E+01	8.77E+01	6.52E+01	2.35E+01
		IFPA	3.33E+01	**3.70E+01**	**3.48E+01**	**8.76E+01**
		SMO	1.22E+01	1.36E+02	5.55E+01	4.28E+01
		AVOA	3.53E+01	4.42E+01	3.79E+01	8.61E+01
*f*_2_	30	FPA	2.67E+02	3.78E+02	3.25E+02	3.38E+01
		IFPA	0.00E-00	0.00E-00	0.00E-00	0.00E-00
		SMO	2.80E+01	3.00E+01	3.72E+01	5.78E+00
		AVOA	**4.17E+01**	**7.38E+01**	**5.26E+01**	**2.45E+01**
*f*_3_	30	FPA	6.68E+00	7.80E+00	6.23E+00	6.98E-01
		IFPA	**7.88E-16**	**7.88E-16**	**7.88E-16**	**0.00E-00**
		SMO	1.82E-01	7.92E-01	2.88E-01	2.32E-01
		AVOA	5.28E+00	6.61E+00	5.60E+00	7.12E-01
*f*_4_	30	FPA	2.09E+00	2.38E+00	2.29E+00	5.37E-02
		IFPA	**0.00E-00**	**0.00E-00**	**0.00E-00**	**0.00E-00**
		SMO	8.78E-03	4.53E-01	7.59E-02	7.46E-02
		AVOA	3.51E-01	8.86E-01	6.73E-01	1.20E-01
*f*_5_	10	FPA	-6.40E+00	-4.98E+00	-5.83E+00	4.36E-01
		IFPA	-8.69E+00	-8.52E+00	-9.25E+00	3.55E-01
		SMO	-8.52E+00	-7.86E+00	-8.97E+00	3.00E-01
		AVOA	**-8.45E+00**	**-7.66E+00**	-6.50E+00	**8.73E-01**
*f*_6_	6	FPA	-4.05345	-3.87203	-4.02038	3.96E-02
		IFPA	**-4.05345**	**-4.05345**	**-4.05345**	5.76E-14
		SMO	-4.05344	-3.88101	-4.02265	3.22E-02
		AVOA	-4.17633	-3.68925	-3.89770	6.38E-02

**Table 3:** Performance comparison of four optimization algorithms of reference function*f*_7_ -*f*_10_.

Function	Dimension	Algorithm	Best value	Worst value	Average value	Standard deviation
*f*_7_	30	FPA	**5.09E+01**	8.92E+01	6.63E+01	2.38E+01
		IFPA	**5.09E+01**	**3.10E+01**	**4.10E+01**	**3.64E+01**
		SMO	**5.09E+01**	3.48E+01	6.12E+01	4.65E+01
		AVOA	**5.09E+01**	4.68E+01	4.19E+01	6.61E+01
*f*_8_	30	FPA	**5.10E+01**	**5.41E+01**	5.28E+01	2.39E+01
		IFPA	**5.10E+01**	5.41E+01	**5.36E+01**	3.17E+01
		SMO	4.15E+01	5.73E+01	5.39E+01	2.65E+01
		AVOA	**5.10E+01**	**5.41E+01**	5.62E+01	**5.43E+01**
*f*_9_	30	FPA	6.14E+01	6.35E+01	6.23E+01	5.60E+01
		IFPA	**6.92E+01**	**4.16E+01**	**4.28E+01**	**4.60E+01**
		SMO	4.19E+01	4.54E+01	4.31E+01	6.66E+01
		AVOA	**6.13E+01**	6.31E+01	6.88E+01	7.09E-01
*f*_10_	30	FPA	-3.16535	-2.87205	-3.16062	3.99E-02
		IFPA	-3.73126	-3.73126	**-3.09535**	**5.83E-14**
		SMO	**-3.10535**	-3.02762	-3.15273	4.48E-02
		AVOA	-3.26871	**-2.97522**	-3.02679	6.95E-02

As can be seen from Table 3, in functions f_7_, f_9_ and f_10_, IFPA performs better than the other three comparison algorithms according to the mean value and standard deviation obtained from 30 independent runs. In function f_7_, although the optimal values of all algorithms are the same, the worst values and average values obtained by IFPA are better than the results of other algorithms. In function f_8_, AVOA, FPA and IFPA have the same optimal value and worst value, while IFPA’s mean and standard deviation are worse than AVOA, but better than FPA. In function f_9_, the best value, worst value, average value and standard deviation obtained by IFPA are all better than the other three algorithms. In function f_10_, although the best and worst values obtained by IFPA are inferior to FPA, the mean value and standard deviation of IFPA are superior to FPA. Therefore, both the optimal value of FPA and the worst value of IFPA are contingent cases, and IFPA is more stable. At the same time, IFPA can obtain better results than the other three algorithms in the optimization stage, and can jump out of the local optimal solution. In particular, in function f_9_, although IFPA is inferior to the other three algorithms at the beginning of optimization, it can quickly jump out of the local optimal in the early optimization stage and continuously develop known regions with its development ability in the whole process to obtain better results.

Therefore, IFPA performs better than the other three comparison algorithms in the four CEC2017 combined benchmark functions, and IFPA greatly improves the performance of FPA.

**Fig 3:**
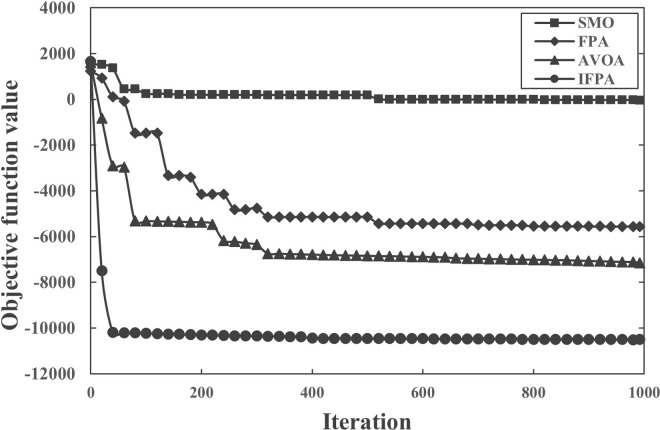
Algorithm convergence curve.

In order to intuitively understand the performance of IFPA, the convergence curve comparison of the algorithm is shown in Fig 3. It can be seen from the convergence diagram of the algorithm that among the test results of the 10 functions, the performance of the three algorithms is almost the same in terms of the optimal value. Only in functions f_2_ and f_5_, the optimal value of the optimization results of the other three algorithms is higher than IFPA, while the rest are opposite. From the average point of view, IFPA performs better than the results of other algorithms except f_2_ and f_5_. In terms of stability, IFPA algorithm with 10 functions performs better than other algorithms. In terms of convergence rate, IFPA, FPA and SMO algorithms all have good convergence rate on 10 benchmark functions, and IFPA has absolute advantage on f_10_ benchmark function.

## 3. IFPA-ELM model

### 3.1. Principle of extreme learning machine

**Fig 4:**
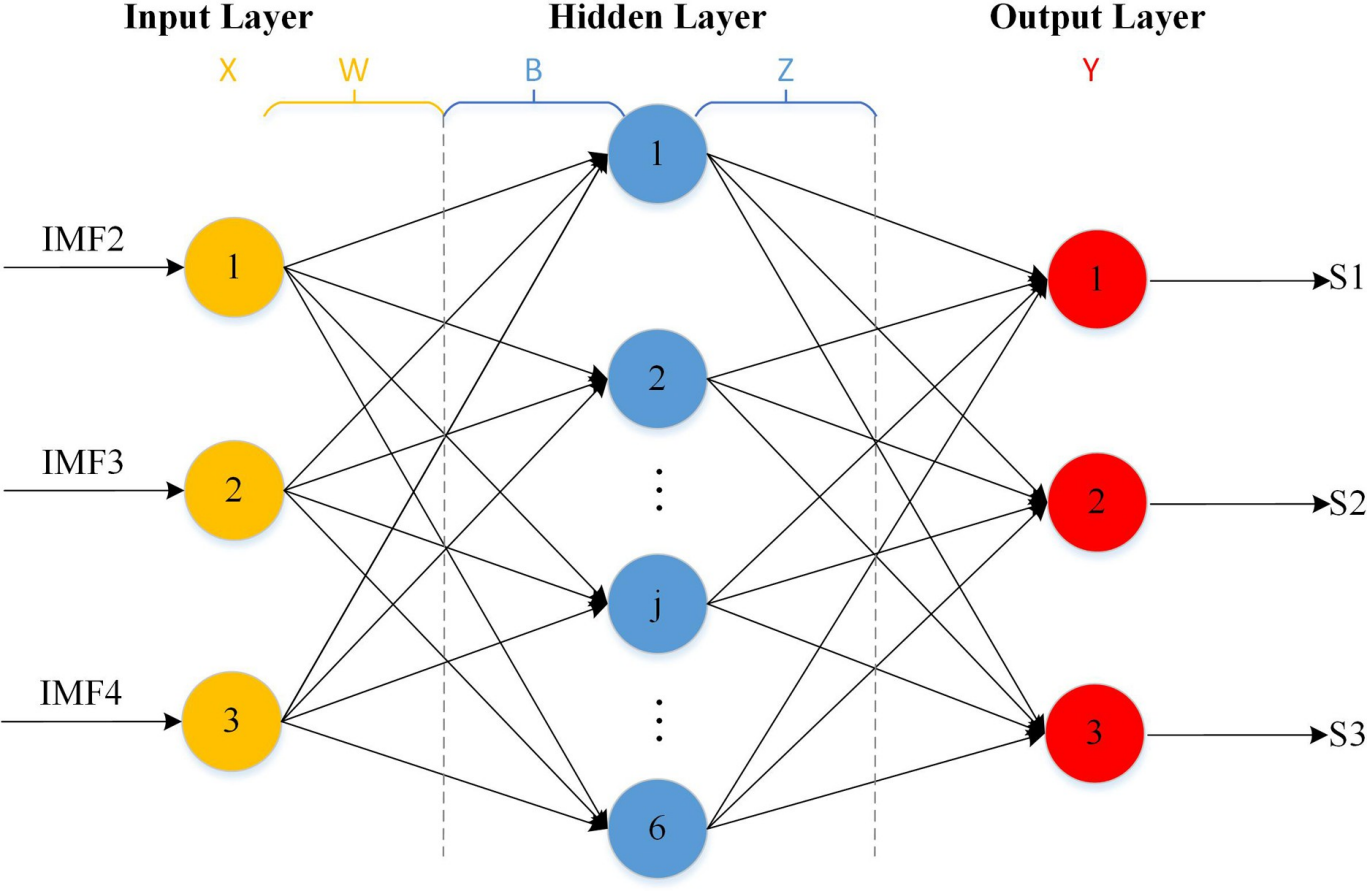
Structure diagram of ELM network.

The ELM is a training algorithm for a single-hidden layer feedforward neural network (SLFN), which was proposed by Huang et al. By randomly assigning the weights and biases of the input layer, ELM determines the weights from the hidden layer to the output layer by computing the Moore–Penrose generalised inverse matrix [35], as shown in Fig 4. The ELM algorithm does not require manual adjustment of the parameters; however, the parameters of the number of hidden layer nodes must be set manually. For a small number of iterations, the learning efficiency and convergence speed are high, which considerably reduces the training time and improves the generalisation performance of the model. However, ELM has poor optimisation accuracy and is prone to falling into a local minimum state. Therefore, the IFPA was adopted for optimisation. A simple statement is as follows:

Suppose N random samples (x_i_, t_i_), where $x_{i}={\left[x_{1}^{i},x_{2}^{i},\cdots ,x_{n}^{i}\right]}^{T}\in R^{n}$ and $t_{i}={\left[t_{1}^{i},t_{2}^{i},\cdots ,t_{n}^{i}\right]}^{T}\in R^{m}$. Here, g(x) is the hidden layer excitation function. Furthermore, the input weight is $W_{i}={\left[w_{i1},w_{i2},\cdots ,w_{in}\right]}^{T}\in R^{n}$, output weight is β, and hidden layer threshold is B. To minimise the output error, the samples are trained with zero error approximation as follows:

Hβ=T (7)

In Eq (7), H is the output matrix of the hidden layer, and T is the expected output [36].

By training the single hidden layer feedforward neural network, the connection weight β can be solved to obtain:

$$\overset{\wedge }{\beta }=H^{+}T$$ (8)

Here, H^+^ is the generalised inverse of the Moore–Penrose of H, and the range of β can be proven to be the smallest and unique [37].

### 3.2. The IFPA-ELM model

**Fig 5:**
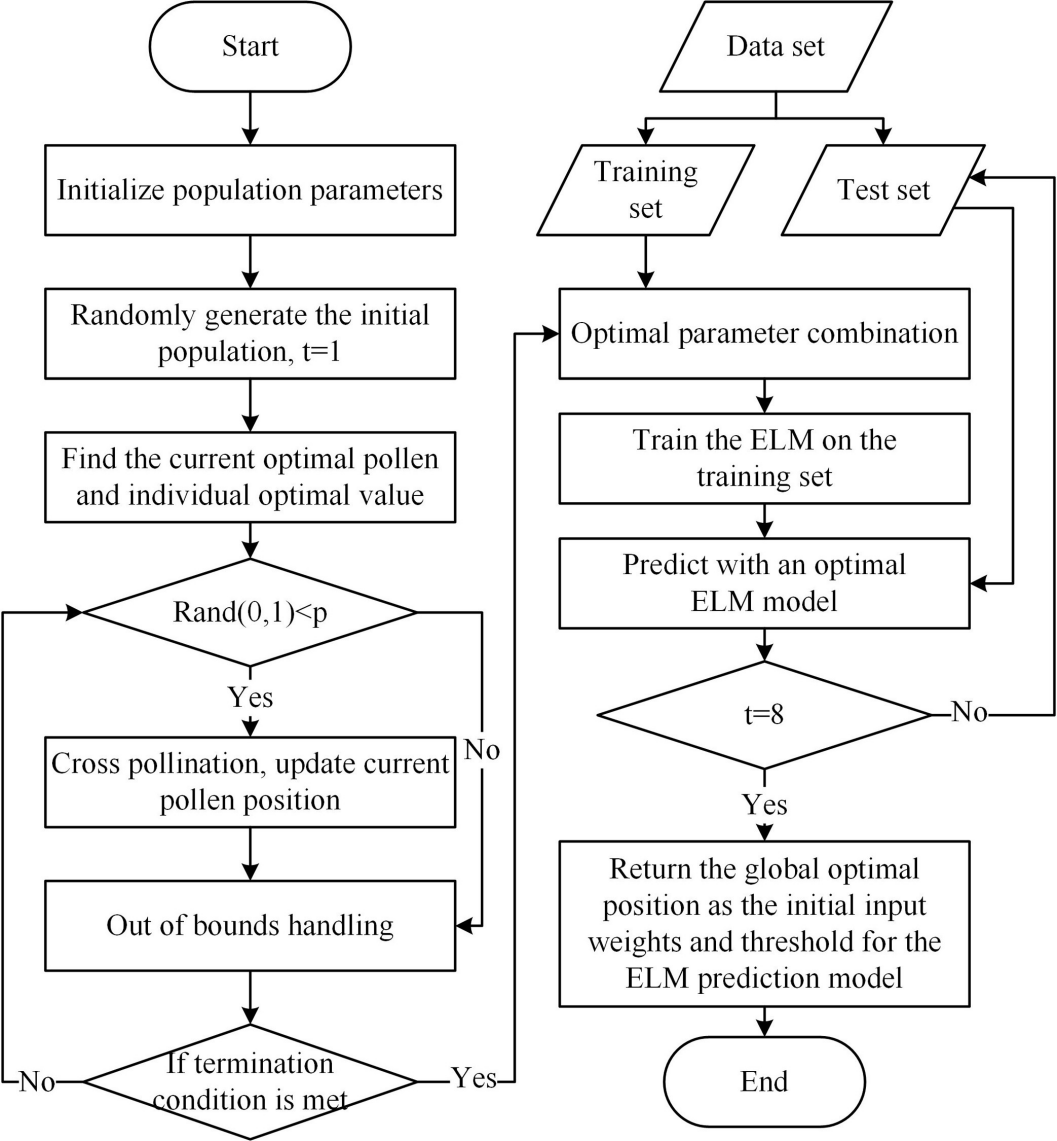
Flowchart of the IFPA-ELM model.

Using the IFPA to optimise the initial weights and thresholds of the ELM algorithm can greatly reduce the variability of the random initial weights and thresholds, and it can enable an intelligent diagnosis of the IFPA-ELM model. The flowchart of the IFPA-ELM model is shown in Fig 5.

## 4. Intelligent identification of the defects in natural gas pipelines

Many gas pipelines in China have been in use for decades, with varying degrees of ageing. Hence, the intelligent identification and classification of pipeline defects is particularly important to ensure the continuous and effective transportation of natural gas pipelines. In this study, we use the Shaanxi-Beijing natural gas pipeline as an example and employed a magnetic flux leakage detector to identify defects in the natural gas pipeline. According to the literature [38], the defect in the outer surface of the detector has a groove width, depth, and length of 0.2, 1, and 2 mm, respectively. Therefore, defects with a depth and diameter of 10 and 2 mm, respectively, can be detected. In this study, the intelligent identification of the pipeline defect signal and classification of its intensity were accomplished by analysing the signal collected from the pipeline.

### 4.1. Feature extraction of pipeline defect signals

**Fig 6:**
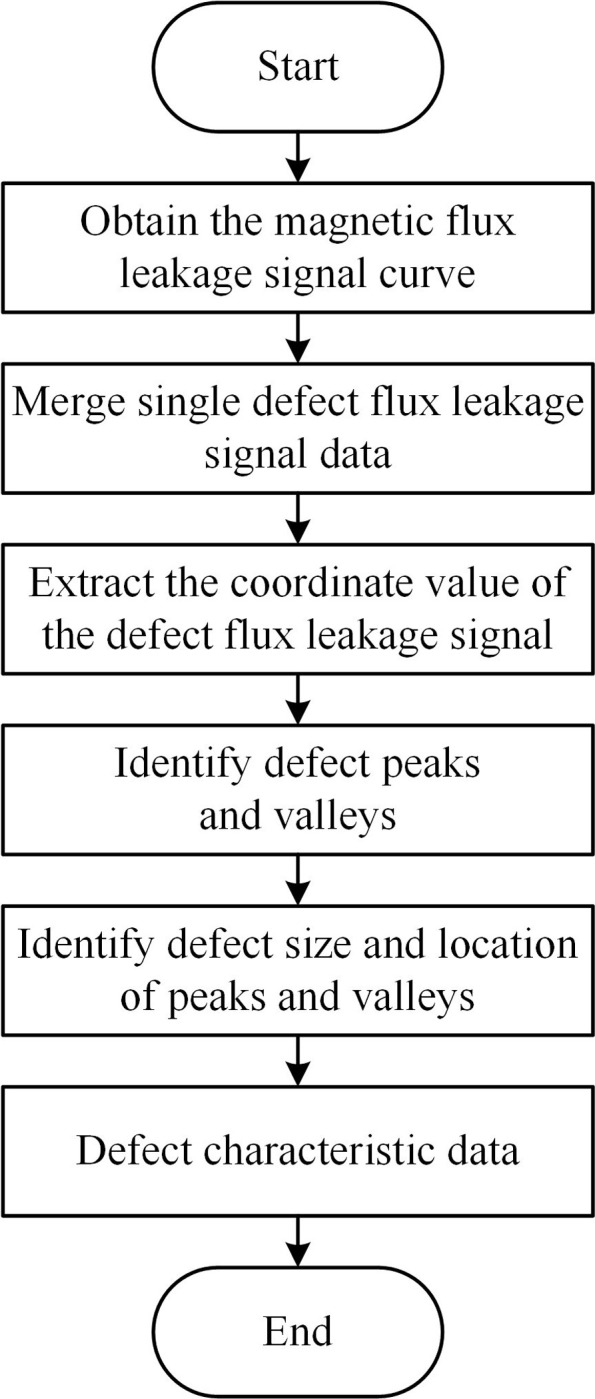
Flowchart of feature extraction of pipeline defect signal.

The MATLAB tool was used to extract the characteristics of the magnetic flux leakage signal pertaining to the pipeline defect, and the flowchart of the process is shown in Fig 6.

We selected the data related to 140 defects. Each defect signal generated several or even dozens of magnetic flux leakage signal waveform curves from which the most representative peak-to-valley curve data were determined, as shown in Table 4.

**Table 4:** Peak-to-valley curve data of a single-peak–double-valley flux leakage signal.

serial number	crest		left trough		right trough	
1	112.96145	5.48500	112.84375	-2.86745	112.99156	-4.36500
2	112.96132	5.44641	112.84390	-2.82767	112.99158	-4
3	112.96113	5.60000	112.84579	-3	112.99160	-3.78266
4	112.96075	5.54587	112.84534	-2.77856	112.99168	-3.40543
5	112.95985	4.54588	112.84156	-2.43882	113.02045	-2.91526
6	112.96090	6.23600	112.84733	-3.30742	113.05071	-2.90665
7	112.96000	3.67632	112.84342	-2	113.06215	-2.55652
8	112.96102	5.52076	112.84015	-1.86956	113.09334	-2.37632

A comparison revealed that the peak and valley data of the sixth waveform curve varies considerably; therefore, the sixth waveform data were selected as the defect signal waveform data. The characteristic signal of each defect can be described by four types of variables: peak–valley value, peak–valley difference, valley–valley difference, and valley–valley value, including two peak–valley values, two peak–valley differences, one valley–valley difference, and one valley–valley value. Therefore, each defect is ultimately described by six characteristic values, as listed in Table 5.

**Table 5:** Six features of pipeline defect signal.

Peak valley1	Peak valley2	Peak valley difference1	Peak valley difference2	Valley difference	Valley value	Defect type
8.1102467	7.1645726	0.017683	0.049263	0.0568	-1.03192	1
31.295769	32.9142961	0.0168	0.01645	0.04176	0.276604	2
11.8621051	11.5689477	0.019	0.0236	0.0478	0.274268	2
9.02767873	13.6389762	0.020711	0.0191	0.037612	4.765112	3
23.4285714	19.5714286	0.023267	0.020937	0.0436	-3.16927	2
7.7735153	6.9615384	0.021	0.0403	0.0513	0.066923	1
2.9370978	3.5458936	0.01545	0.0223	0.04864	0.701532	2
4.9263736	6.4210526	0.01494	0.01296	0.0279	1.306892	2
3.2971819	5.717673	0.0087	0.0189	0.0376	2.7	2
5.72	4.76	0.01738	0.010475	0.01785	-1.72	3
5.228491	1.6096956	0.01567	0.0073	0.02295	-3.5078	3
7.5316789	7.9	0.024093	0.015073	0.040965	-0.59158	2
…	…	…	…	…	…	…
3.6875	3.975	0.013892	0.0142	0.025956	0.1985	2
2.6580589	3.2765706	0.013148	0.016965	0.030192	0.529412	2
6.0764	7.0768	0.015783	0.018576	0.02918	1	2
8.598245	9.6104218	0.0164	0.017638	0.032815	0.951286	2
1.89146397	2.7105243	0.011521	0.018753	0.030762	0.793529	3
3.9625274	4.10763562	0.026014	0.017789	0.032707	0.236383	2
4	4.62	0.02435	0.017653	0.031652	0.56	2
3.521411	3.6	0.021149	0.014812	0.02498	-0.11112	3
3.39	4.46	0.0171	0.0237	0.0289	1.05	2
9.92465711	12.3796623	0.014689	0.015632	0.029332	2.315761	2

The normal pipeline, pit defect, and crack defect signals were selected as the set of pipeline defect signals, namely, *S* = {*S*_1_, *S*_2_, *S*_3_}. Pipeline defect signals inevitably contain a certain amount of noise. A traditional noise reduction method, such as wavelet noise reduction, is not ideal because it easily results in incomplete signal noise reduction. Singular values easily result in signal offset, whereas the integrated empirical mode decomposition method adds white noise. This increases the number of intrinsic mode functions (IMFs) obtained via decomposition, making it unsuitable for identifying and classifying pipeline defects. The empirical mode decomposition method, which we selected for this study, can adaptively decompose the signal and extract the characteristic parameters related to the pipeline defect.

**Fig 7:**
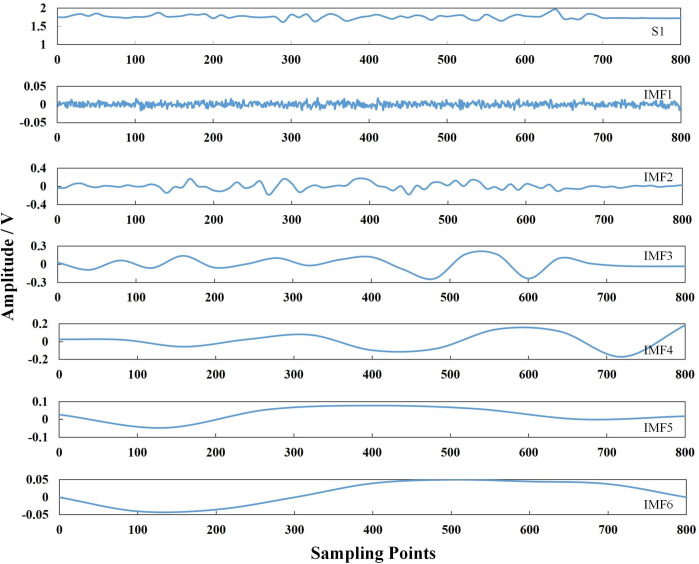
EMD diagram of a normal pipeline signal S1.

**Fig 8:**
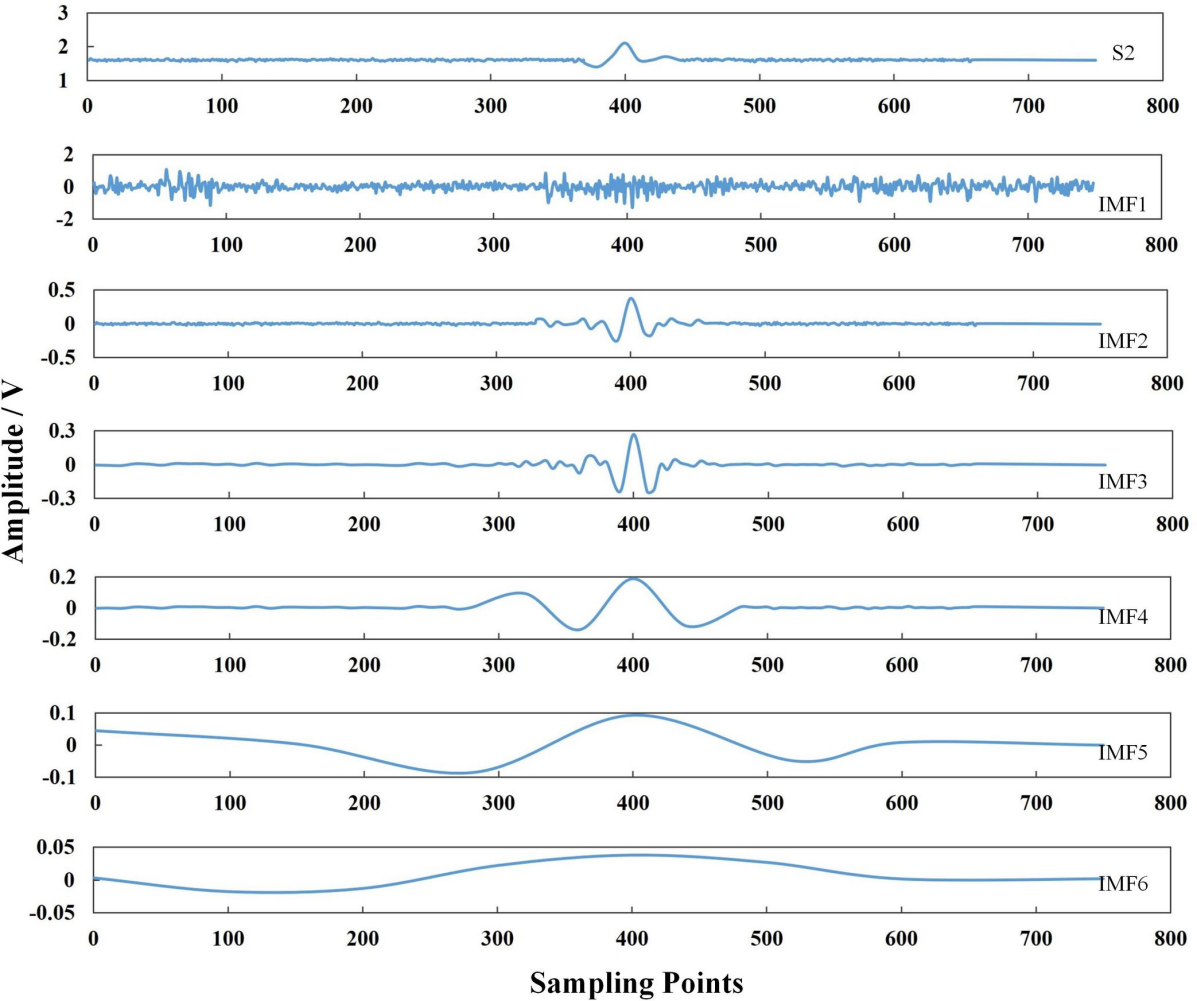
EMD diagram of a pipeline pit defect signal S2.

**Fig 9:**
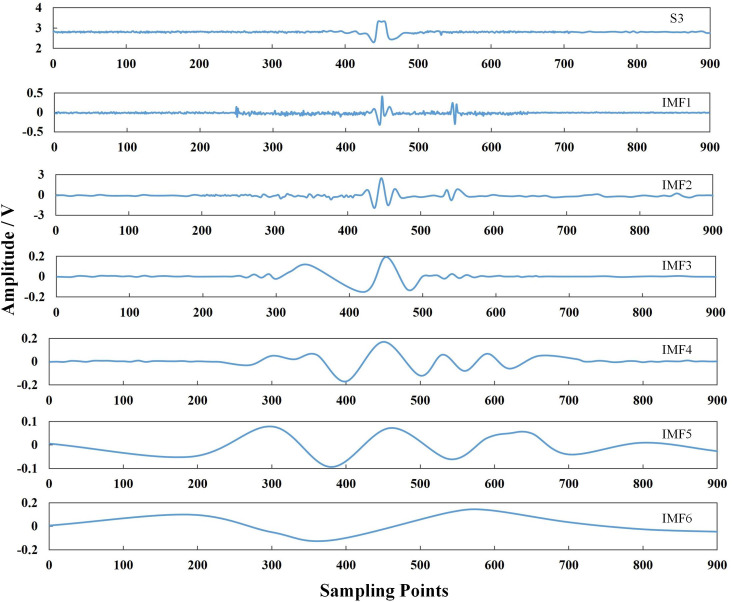
EMD diagram of a pipeline crack defect signal S3.

Empirical mode decomposition (EMD) is an adaptive decomposition algorithm, which can decompose complex signals into a linear superposition of multiple IMFs [39]. Figs 7–9 show the signal decomposed into single-component signals *imf*_*1*_, *imf*_*2*_, *imf*_*3*_ . . . *imf*_*6*_, and using the following equation:

$$\rho \text{=}\frac{\sum_{k=0}^{\infty }imf_{i}\left(k\right)x\left(k\right)}{\sqrt{\sum_{k=0}^{\infty }imf_{i}^{2}\left(k\right)\sum_{k=0}^{\infty }x^{2}\left(k\right)}}$$ (9)

**Fig 10:**
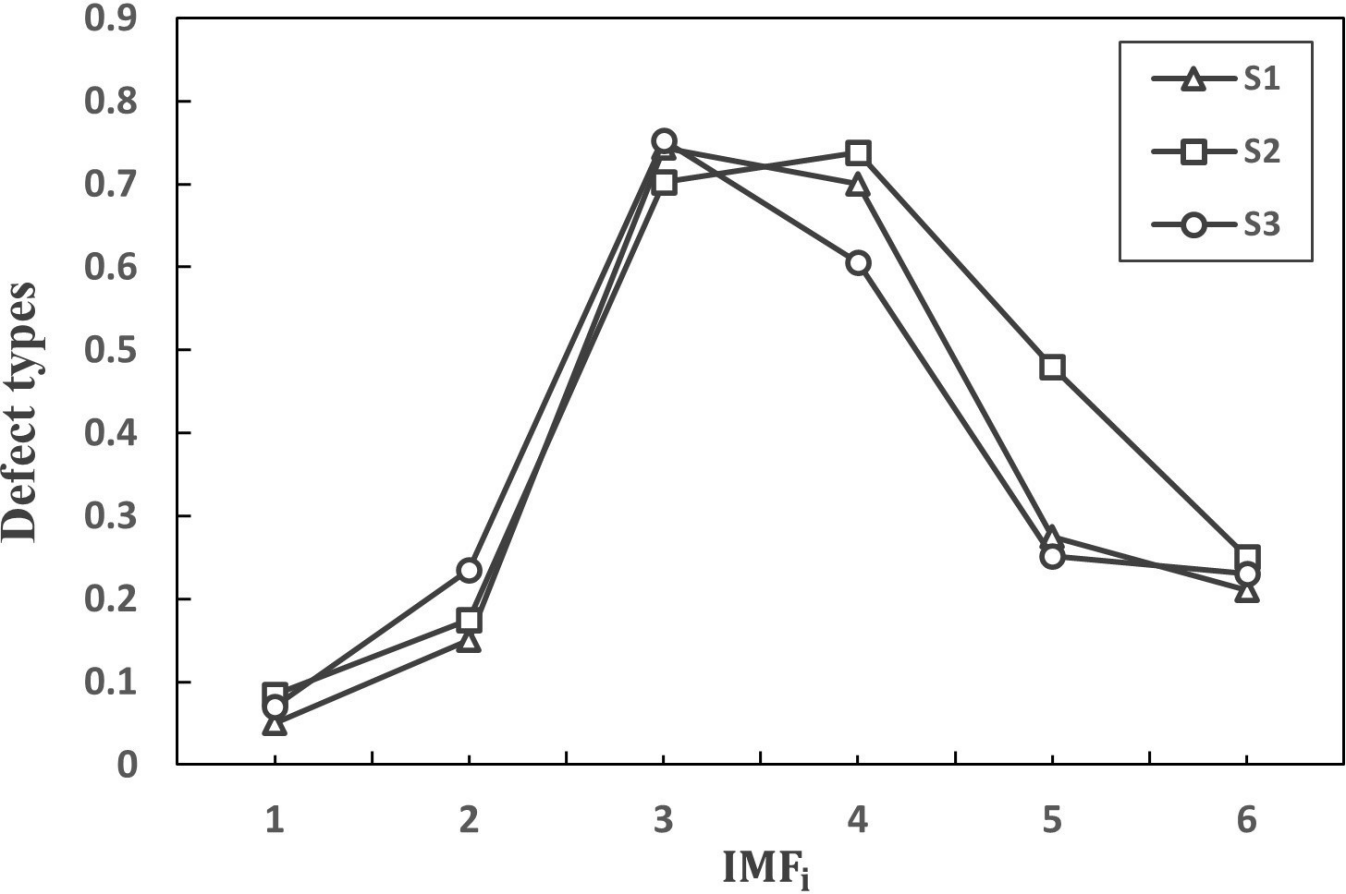
Correlation coefficient plot of each IMF component and the original signal.

Eq (9) was used to calculate the correlation coefficient between each component *imf* decomposed via EMD and the original signal [40]. The correlation coefficients of S_1_, S_2_ and S_3_ and their respective IMF (Fig 10) were derived by calculating the similarity between each IMF component and the original signal.

Among them, *imf*_*i*_ is the *i*^th^ IMF constituent, *x* represents the actual signal, and *ρ* represents the correlation coefficient between the *imf*_*i*_ component and original signal *x*.

The correlation coefficients of the IMF components with raw data are shown in Table 6. The correlation coefficients of *imf*_*2*_, *imf*_*3*_, and *imf*_*4*_ components are large, at 0.8620, 0.7253, and 0.5708, respectively. The *imf*_*2*_, *imf*_*3*_, and *imf*_*4*_ components are the real components of the signal, and *imf*_*1*_ with the smaller correlation coefficient is removed; *imf*_*1*_, *imf*_*5*_, *imf*_*6*_ are pseudocomponents. Therefore, we selected *imf*_*2*_, *imf*_*3*_, and *imf*_*4*_ to extract the feature parameters.

**Table 6:** Table of IMF components in relation to raw data.

weight	IMF_1_	IMF_2_	IMF_3_	IMF_4_	IMF_5_	IMF_6_
Correlation coefficient	0.0375	0.8620	0.7253	0.5708	0.0679	0.08859

The sample entropy is the weight of the probability of a system to generate new patterns from the point of view of time series complexity; it quantitatively describes the complexity and regularity of the system [41]. The sample entropy is used to evaluate the complexity and regularity of time series. In this study, the randomness of the identification of pipeline defect signal is evaluated according to the sample entropy of each IMF component to determine the difference between the normal and defect signals of the pipeline. The lower the sample entropy, the higher is the time series self-similarity; the higher the sample entropy, the higher is the time series complexity. The determined value of the sample entropy for the sequence length L is given by Eq (10).

$$SampEn\left(m,t,L\right)=-\text{ln}\frac{B^{m+1}\left(t\right)}{B^{m}\left(t\right)}$$ (10)

Here *m* = 2, *t* = 0.2 std, where std represents the standard deviation of the raw discrete sequence, and *B* is the average.

The sample entropy was selected from 140 group samples to obtain pipeline defects, as listed in Table 7, including the inevitable presence of noise and a certain extent of low-intensity signal interference. While detecting a pipeline with defects, the strength of the defect signal is considerably greater than that of the jamming signal, resulting in a decrease in the entropy of each IMF sample. Therefore, the IMF sample entropy is selected as the feature vector of pipeline defects to perform defect pattern recognition on the pipeline state.

**Table 7:** Sample entropy of each IMF.

Defects	IMF_1_	IMF_2_	IMF_3_	IMF_4_	IMF_5_	IMF_6_
S_1_	0.0353	0.8228	0.6586	0.4241	0.0036	0.0024
S_2_	0.0235	0.8639	0.7438	0.5957	0.0069	0.0051
S_3_	0.0836	0.7852	0.7362	0.5147	0.0026	0.0015

### 4.2. Identification of the defect signals from natural gas pipelines

The test results of the six benchmark functions show that the IFPA exhibits superior performance. In addition, the combination of IFPA optimisation with ELM, maximises the accuracy and speed of the algorithmic model. These results confirm the suitability of the proposed IFPA to detect and identify signals associated with the defects in a natural gas pipeline. The features of the defects were extracted from the 140 defect samples selected as training samples. Each defect had six corresponding attribute features. According to the literature [42], when the number of neurons in the hidden layer is the same as the number of samples in the training set, the model of the algorithm can approximate all training samples without error. Therefore, 50 neurons were used in the hidden layer for simulation. According to the roughness of the defect attribute data, the pipeline defects were divided into three types: normal state, signal and pit defect, and crack defect signals.

The ELM, FPA-ELM and IFPA-ELM algorithms were used to distinguish the pipeline state, and the basic parameters of the FPA and IFPA were assigned to the same value. The specific parameters are listed in Table 8. The prediction results of the two groups of samples in the three states of the pipeline are listed in Table 9. The findings show that the original ELM algorithm erroneously identified two pipeline defects, and the IFPA-ELM algorithm incorrectly classified one pipeline defect. Despite these errors, the FPA-ELM algorithm significantly improves the recognition accuracy of the algorithm.

**Table 8:** Main parameter settings of the optimisation algorithm.

Algorithm	Parameters	values
FPA	Switch probability	0.8
	Levy flight *λ*	1.5
	Scaling factor	0.01
IFPA	Switch probability	0.8
	Levy flight *λ*	1.5
	Scaling factor	0.01
	Mutation constant *ε*	0.9
SMO	Perturbation rate	0.92
	Maximum fitness	0.885
	Inertia weight	0.9
AVOA	Step size	0.2
	Exploration probability	2.5
	Optimal position	0.6
	Suboptimal position	0.4
	New location	0.6

**Table 9:** Output results based on three algorithms.

Algorithms	S_1_	S_1_	S_2_	S_2_	S_3_	S_3_
ELM	**0.9971**	**0.9925**	0.2619	0.0091	0.0326	0.0009
	0.0001	0.3512	**0.9987**	**0.9489**	**0.9972**	**0.7563**
	0.0869	0.0000	0.0047	**0.0419**	**0.0098**	0.2193
FPA-ELM	**0.9968**	**0.9969**	0.0000	0.0000	0.0000	0.0000
	0.0000	0.0000	**1.0000**	**1.0000**	0.2019	**0.6019**
	0.0056	0.0051	0.0000	0.0000	**0.5162**	**0.4115**
IFPA-ELM	**1.0000**	**0.9989**	0.0000	0.0003	0.0000	0.0000
	0.0000	0.0612	**1.0000**	**1.0000**	0.0036	0.8193
	0.0000	0.0000	0.0000	0.0000	**1.0000**	**0.9695**

The recognition rate of the 140 defect samples is presented in Table 10. Corresponding to the signal of the normal pipeline, the recognition rates of the three algorithms are all 100%, whereas the ELM recognition rates of the two defect signals are 72% and 56%, respectively. Additionally, the recognition rates of the FPA-ELM algorithm are 89% and 82%, respectively. Considering that the ELM algorithm readily converges to local minima, FPA was used for optimisation. Although the recognition rate improved, it was still lower than 90%, indicating that the FPA algorithm still required further improvement. The efficiency and accuracy of the IFPA-ELM algorithm are considerably higher than those of the ELM and FPA-ELM algorithms.

**Table 10:** Comparison of the identification rates of defective samples.

Types	ELM	FPA-ELM	IFPA-ELM
S1	100%	100%	100%
S2	72%	89%	97%
S3	56%	82%	96%

## 5. Conclusion and future works

The accurate identification of defects in natural gas pipelines is important to ensure their continuous, efficient, and stable operation. The ELM algorithm has a high learning rate and good generalisation performance; however, the convergence accuracy of this algorithm is poor, and it easily converges to local minima. The introduction of IFPA improves the convergence accuracy and searchability of ELM, and it outperforms traditional intelligent algorithms such as SMO and AVOA.

The relatively poor optimisation ability of FPA motivated us to improve this algorithm by combining the adaptive adjustment based on the transformation probability and Gaussian mutation strategy. The combination of these two techniques enables a higher diversity of the population and an appropriate balance of global and local searches. The Gaussian mutation strategy improves the exploration ability of the algorithm. Six classical benchmark functions were used to test and verify the superiority of the improved algorithm and to compare its performance with SMO, AVOA, and FPA. The main contributions of this study are an adaptive adjustment of the conversion probability, introduction of the Gaussian mutation strategy into FPA, optimisation of the initial input weight and threshold of ELM to stabilise and improve the explorative ability of the algorithm, and higher efficiency and accuracy of pipeline defect identification.

The proposed IFPA-ELM combines the advanced IFPA with the ELM algorithm to identify pipeline defects. The proposed algorithm was evaluated by comparing its performance in terms of pipeline defect identification with that of the ELM, FPA-ELM, and IFPA-ELM algorithms. The results showed that, compared with the other algorithms, the IFPA-ELM model identifies defect signals more accurately. Finally, it is concluded that the recognition rates of various pipeline defects by the IFPA-ELM algorithm were to be 97% and 96% respectively. The recognition rates of the proposed algorithm are 34% and 13% higher than those of the FPA and FPA-ELM, respectively.

However, the following limitations of the proposed IFPA-ELM model must be addressed: Because IFPA is a combination of multiple algorithms, the time cost of computation is increased, and this aspect requires further optimisation. In addition, the constant value of the conversion probability in the algorithm greatly affects the performance of the algorithm. Hence, the performance of the algorithm could be assessed by determining the range of conversion probability values or the common adaptive value to set the appropriate value or adjust the balance between global and local search. Finally, the algorithm could be applied to complex engineering optimisation problems in various fields for further research in future.
